# Genomic and Proteomic Biomarker Discovery in Neurological Disease

**DOI:** 10.4137/bmi.s596

**Published:** 2008-02-09

**Authors:** RiLee H. Robeson, Andrew M. Siegel, Travis Dunckley

**Affiliations:** 1 Neurogenomics Division, Translational Genomics Research Institute, Phoenix, Arizona 85004

**Keywords:** biomarker, neurological disease, neurodegeneration, alzheimer’s disease, parkinson’s disease, genomics, proteomics, gene expression profiling, SNP, aCGH

## Abstract

Technology for high-throughout scanning of the human genome and its encoded proteins have rapidly developed to allow systematic analyses of human disease. Application of these technologies is becoming an increasingly effective approach for identifying the biological basis of genetically complex neurological diseases. This review will highlight significant findings resulting from the use of a multitude of genomic and proteomic technologies toward biomarker discovery in neurological disorders. Though substantial discoveries have been made, there is clearly significant promise and potential remaining to be fully realized through increasing use of and further development of -omic technologies.

## Introduction

The ultimate goals for research focused on complex human diseases are to either prevent or to cure the diseases. These are ambitious goals that will be greatly facilitated by the identification of new biomarkers that can serve as novel diagnostic or prognostic indicators of disease course, that can be used as surrogate disease markers to track the efficacy of novel treatment strategies, or that may provide new targets for the treatment of the diseases.

Neurological disorders present multiple unique challenges to biomarker discovery that are more extensively reviewed elsewhere (Dunckley et al. 2005). Briefly, the essential and vital role that the brain and central nervous system play in all aspects of life ensures the lack of availability of tissue at the site of pathology for most neurological disorders. Additionally, the ante mortem clinical diagnostics, although continually improving in most neurological diseases, remains problematic. In many diseases, such as Alzheimer’s disease and Parkinson’s disease, definitive diagnosis occurs only at autopsy with sometimes as many as 20% of cases being found to have been clinically misdiagnosed. Reliable genetic or protein based biomarkers specific for these diseases would clearly be useful for earlier and more accurate diagnoses and treatment interventions. Lastly, there are few animal models fully representative of the diseases that can be used for validation of candidate biomarkers. This is likely due in part to the increased complexity of the human brain, of human behavior, and the possibility that neurological diseases, many of which develop later in life and require an aging component, are difficult to recapitulate in shorter lived animals.

High-throughput technologies for studies of the human genome and proteome are advancing at a rapid pace and are increasingly being applied toward the study of neurological diseases to help overcome some of these hurdles. This review will focus on the application of these current high-throughput strategies to biomarker discovery in neurological disease.

## Genomics

The study of genomes, referred to as ‘genomics,’ has made substantial progress toward our understanding of human disease and promises to direct the future of medicine toward more personalized diagnostics and therapeutics based on an individual’s specific genetic variations and predispositions to disease. The majority of complex human diseases have a genetic component that has historically been difficult to uncover using traditional genetic methods. Indeed there are numerous examples of the use of genomic technologies to identify novel biomarkers for multiple neurological diseases, including Alzheimer’s disease, Parkinson’s disease, Autism and Huntington’s disease among others (Borovecki et al. 2005; Coon et al. 2007; Dunckley et al. 2005 and 2007; Fung et al. 2006; International Multiple Sclerosis Genetics Consortium et al. 2007; Lee et al. 2007; Maraganore et al. 2005; Papapetropoulos et al. 2007; Papasssotiropoulos et al. 2006; Reiman et al. 2007; Scherzer et al. 2007; Schymick et al. 2007; Tang et al. 2005; Ulmann et al. 2007; van Es et al. 2007). However, critical findings that have been translatable to clinical application seem to be intermittent at best due to the challenges facing neurological research as previously discussed.

The genetic underpinnings of mendelian forms of disease have been pieced together using familial genetic approaches, such as linkage studies, where whole families are studied to find commonly inherited mutations associated with disease. This approach has been successful in identifying the genetic causes of some very prominent familial forms of neurodegenerative diseases, such as Alzheimer’s disease (AD) and Parkinson’s disease (PD). However, even with these common disorders, familial cases account for a small fraction of individuals afflicted with the disease. A significant challenge lay both in developing biomarkers for and understanding the causes of the much more prevalent sporadic forms of complex neurological diseases, such as late onset AD (LOAD). In the following sections, the main genomic technologies currently being applied to this problem will be described and recent findings discussed. These areas include array CGH (array comparative genomic hybridization), gene expression profiling, whole genome association studies using single nucleotide polymorphisms (SNPs), and, more toward the future, whole genome resequencing to identify causal disease-associated DNA sequence variants.

### Array CGH

On a functional level, array comparative genomic hybridization (aCGH) measures copy-number variations at multiple locations in the genome simultaneously (Barrett et al. 2004) by comparing DNA content from two differentially labeled genomes: one being the patient (or test) genome and the second being the control (or reference) genome (Bejjani and Shaffer, 2006). Both genomes are isolated, fluorescently tagged with different fluors, and then competitively hybridized to a solid surface where cloned or synthesized DNA fragments corresponding to defined segments of genomic DNA have been bound (Bejjani and Shaffer, 2006). Once an image of the slide has been captured, the level of fluorescence signals from each DNA sample for each target placed on the array is calculated (Bejjani and Shaffer, 2006). The relative fluorescence intensity of the test genome compared to the reference genome provides a measure of the number of DNA copies in that region of the genome in disease versus control. Using this technology one can show changes such as deletions, duplications, or amplifications at any locus that is represented on the array (Bejjani and Shaffer, 2006). Unlike FISH (fluorescence *in situ* hybridization), which is limited in the number of loci that can be simultaneously interrogated, aCGH is able to detect DNA copy number changes at hundreds of thousands of loci in a genome in a single experiment and thus provides a far more rapid and facile method for detecting genomic copy number variations associated with disease.

Originally developed as a research tool to identify copy number variations in cancer (Barrett et al. 2004; Bejjani and Shaffer, 2006), aCGH is one area that is still in its beginnings with respect to applications in neurological research. It is typically used for discovering disease-associated loci and developing diagnostic or therapeutic targets in cancer and developmental disorders (Barrett et al. 2004). Only a small number of studies have recently explored the application of aCGH to the study of neurological disease. These efforts have focused primarily on mental retardation, autism and various forms of epilepsy (Bonaglia et al. 2005; Ulmann et al. 2007; Toruner et al. 2007; Edelmann et al. 2007). Neurodegenerative diseases specifically have yet to be researched. This may be due in part to the lack of high quality tissue samples from affected disease areas, a key element for reliable aCGH results. Additionally, with the notable exception of α-synuclein locus triplication in a familial form of Parkinson’s disease (Singleton et al. 2003), there is not much evidence to suggest that structural genomic abnormalities or changes in copy number broadly underly neurodegenerative diseases. This may however simply reflect a lack of sufficient investigation in this area. However, as tissue collection methods are improved and standardized, aCGH technology may yet reveal additional significant alterations associated with neurological disease.

Array CGH has been suggested as a potential diagnostic tool to personalize treatment strategies. Recently, aCGH has been used to detect copy number alterations in the *PLP1* region for prenatal diagnosis of Pelizaeus-Merzbacher disease (PMD) (Lee et al. 2005), a rare X-linked dysmyelinating disorder of the central nervous system (Garbern et al. 1999; Inoue, 2005). Genomic duplications of the *PLP1* (proteolipid protein 1) gene is one of the main causes of PMD, and has been used to molecularly diagnose the disease by interphase FISH and quantitative multiplex PCR methods using blood samples in children and adults (Lee et al. 2005; Inoue et al. 1996). It is also well established in prenatal diagnosis by using either amniotic fluid or chorionic villus sampling (Lee et al. 2005; Woodward et al. 1999; Inoue et al. 2001; Regis et al. 2001). Array CGH has been used to successfully detect *PLP1* copy number in the developing fetus and provides the noted benefit of being more efficient than one FISH experiment (Lee et al. 2005). The specific size of genomic duplication can also be determined using aCGH provided that the probe density is sufficient, something that is more labor intensive using FISH. One limitation of aCGH however is that it cannot detect other rearrangements, such as inversions or balanced translocations. Nevertheless, aCGH provides a rapid diagnostic for PMD and establishes the utility of this technology for assisting in the diagnosis of neurologic diseases.

This paradigm of using aCGH as a diagnostic tool may yet prove useful in other neurological diseases as emerging evidence is beginning to link copy number alterations to complex neurological disorders. For example, Ulmann et al. recently identified reciprocal duplications and deletions on chromosome 16p13.1 that are associated with autism and/or mental retardation (MR) (Ulmann et al. 2007). With further validation of this genetic association, aCGH may provide a more accurate diagnostic tool for a subset of autistic individuals. This is particularly important in autism where the difficulty in making a definitive clinical diagnosis often delays administration of appropriate behavioral therapy, which is substantially more effective when initiated early in life.

It should be noted that changes in copy number are remarkably common in the human population, irrespective of association with disease (Bejjani and Shafer, 2006). Therefore to identify a copy number change that is sensitive and specific for a complex neurological disease, such as autism or Alzheimer’s disease, will likely require the initial study of thousands of patients and controls to derive sufficient power to detect a consistent and significant association with disease, if such an association exists.

### Gene expression profiling

Gene expression microarrays are used to rapidly assess the expression of thousands of genes in a single experiment, generating specific “expression profiles” of normal and disease states. There are two basic types of microarrays commonly used (Schena et al. 1995; Shalon et al. 1996; DeRisi et al. 1996). Both fix DNA probes to a solid surface. However, one type of array uses cDNA for probes (usually 200–2000 nucleotides long) and another uses oligonucleotides (usually 25–70 nucleotides long). Labeled cRNA (or cDNA) is then hybridized to the array and binds to specific regions of the array that contain probes of complementary sequence. Expression levels are calculated based on the quantity of signal coming from each probe location, which is related to the amount of labeled cRNA or cDNA bound to the array at that location. Oligonucleotide arrays have greater specificity, as well as the ability to distinguish between splice variants if probe sets are designed to cover different exons, as is the case specifically for exon arrays. Although cDNA arrays have higher sensitivity resulting from the increased probe length, they often cannot be designed to distinguish between splice variants because the length of the DNA probes usually span splice junctions.

A typical experiment will compare tissue from a healthy control to tissue from an individual affected by a specific disease. The mRNA is isolated, amplified, converted to cDNA, and labeled. Samples are then hybridized to the probes on the arrays, which correspond to regions of specific genes of interest, and imaged. Comparison of the expression profile for the healthy tissue to that from the disease tissue will identify specific gene dysregulation correlated with the disease of interest. This disease related expression signature provides information about the underlying cellular dysfunction involved in disease pathogenesis, or could be used as a diagnostic gene expression signature to help guide treatment options.

Given the large number of genes interrogated in each experiment, reproducibility is a key issue. Multiple reports have addressed this issue and results show that intra-platform reproducibility is significantly higher than inter-platform reproducibility (Hollingshead et al. 2005; Shi et al. 2006; Canales et al. 2006). This likely results from the fact that different manufacturers use unique sets of probe sequences, each with different hybridization efficiencies. Thus for the most consistent results, repeated measures should use the same platform for all comparisons. For identifying validated correlates of disease that are indicative of the underlying disease-relevant mechanisms, typically 15 to 20 case samples and 15 to 20 control samples are needed (MacDonald et al. 2001; Dunckley et al. 2006). However, due to disease heterogeneity, varied environmental influences and inter-individual variability, development of specific and sensitive diagnostic expression signatures requires a larger number of samples, typically from 30 to 100 or more cases and controls (Shirahata et al. 2007; Magic et al. 2007; see also Burcszynski et al. 2005 and references therein). Of course, more samples will increase the likelihood of generating more specific and sensitive expression signatures diagnostic of disease.

Gene expression profiling has been very useful for identifying the underlying cellular changes involved in idiopathic and multifactorial diseases, including neurodegenerative disorders such as AD and PD (Papapetropoulos et al. 2007). One of the advantages of gene expression profiling is its applicability to any tissue sample that contains intact mRNA, such as brain tissue or blood. Importantly, identification of disease-specific differences in gene expression in blood would provide a facile diagnostic biomarker for neurological disease, although thus far no such reliable biomarker has been forthcoming.

Currently there is a large amount of ongoing research using gene expression profiling in neurological diseases. However, the context of biomarkers seems to have the most clinical potential when looking primarily at blood samples. Recently, Sharp et al. identified sensitive genomic profiles for human individuals who suffered an ischemic stroke that were also specific for predicting strokes (Sharp et al. 2006). In a previous study, this group also looked at individuals with Down syndrome and found that they could see different expression profiles in the blood that reflected different phenotypes appearing within the disease (Tang et al. 2004). Multiple differentially expressed genes have also been identified in natural killer and CD8 cells of individuals with Tourette syndrome (TS) (Tang et al. 2005; Sharp et al. 2006).

Another recent study looked at potential diagnostic gene expression markers for Parkinson’s disease (PD) in blood (Scherzer et al. 2007). Using a two-stage experimental design with a training and validation population, a set of dysregulated genes was significantly associated with risk of PD (odds ratio = 5.7). The set of genes identified predict PD significantly more effectively than current prediction methods, which include the risk factors of age and sex (Scherzer et al. 2007). The genes within this set do not correlate to a single pathway, but all are known to be expressed in the human brain (Rebhan et al. 1998; Kalchman et al. 1996). Also, many of the genes seem to be previously identified or associated with PD or with a major process that could be contributing to PD. With no current laboratory test available and early detection of PD being clinically difficult, these findings may advance the development of diagnostic biomarkers for PD. Furthermore, it is striking that dysregulated genes identified in peripheral blood samples in this study reflect many of the known pathogenic mechanisms occurring in the brain of patients with PD. This suggests that in some cases insights into biological pathogenesis of neurological diseases may be gained from identifying aberrations in blood samples, despite the presence of an intact blood-brain barrier.

Another interesting study examined gene expression profiles in a group of patients suffering from Huntington’s disease (HD) (Borovecki et al. 2005). These authors identified a subset of mRNAs that successfully distinguished controls from pre-symptomatic individuals with the HD mutation and from symptomatic HD patients (Borovecki et al. 2005). Thus, these dysregulated genes were able to discriminate the different stages of the disease, suggesting that this marker set is able to monitor disease progression. In addition, numerous genes from this biomarker set were also dysregulated in HD post-mortem frozen brain samples. Thus, this example and the one above for PD suggest that dysregulation in blood may reflect dysregulation occurring in the brain during the course of neurodegenerative diseases. For HD, the correlation between brain and blood may mechanistically result from mutated huntingtin protein similarly affecting brain and blood targets. These provide useful examples of the study of peripheral tissues to gain insights into the pathology occurring in a less accessible, but more functionally relevant tissue, the brain.

The findings from these studies and many others illustrate the potential and evolving capability of gene expression profiling to determine distinct profiles attributed to different phenotypes in a disease, to identify individual subtypes of complex diseases like Tourette Syndrome, autism and others that possibly have central environmental and genetic factors contributing to the disease (Sharp et al. 2006), and also to develop a way to monitor disease progression, early recognition and correlate activities in the brain to markers in blood.

### Single nucleotide polymorphisms

Any two humans are 99.9% identical at the DNA sequence level. However, it is the 0.1%, or ~1 nucleotide in 1,000, variation that defines our uniqueness as well as our differential susceptibility to diseases. Single Nucleotide Polymorphisms (SNPs) represent single base pair differences between individuals at a specific location in the genome. There are an estimated 10 million SNPs that differ between individuals in the population (Ardlie et al. 2002; Carlson et al. 2003; Kruglyak and Nickerson, 2001). A comprehensive cataloguing effort of these variable positions through multiple efforts, including the SNP consortium (TSC), the Celera sequencing effort, and the HapMap project now allows the utilization of these SNPs to interrogate the specific combinations of SNPs that, when co-inherited, predispose to any human trait. Only recently have genomic advances matured to the point that genotyping large numbers of SNPs to address the genetics of complex disease is becoming possible.

SNPs are grouped into three functional classes. The first group contains the classically defined Mendelian inherited single base-pair mutations. These are single SNPs occurring in the coding region of genes that alter the protein sequence of the gene product or that result in premature truncation of the encoded protein. These are predicted to have strong functional effects on their own. The second class of SNPs, referred to as functional SNPs, consist of those that have subtle effects on gene function or expression, contributing to disease only when occurring in the context of additional genetic variants or environmental influences. The third category consists of nonfunctional SNPs, those that are functionally completely silent, but may nevertheless be of interest due to genetic linkage to a nearby functional DNA sequence variant. The majority of SNPs fall into this category.

Functional SNPs are of particular interest in the study of common and complex neurological disorders since they are thought to occur at high frequencies in the general population and to result in disease when occurring in specific combinations. Recent advances in high-throughput SNP genotyping technologies provide an opportunity to identify these SNPs in large case-control association studies of complex neurological diseases. SNP analysis also enables more rapid identification of Mendelian inherited diseases through linkage analyses, in some cases Condensing The previous time-frame for disease gene identification from several years for traditional approaches to less than a week for high-throughput SNP based assays (Puffenberger et al. 2004). Here we discuss applications to complex neurological disorders as this class comprises the majority of neurological disease.

## Analyzing Complex, Multigenic Disorders

SNP genotyping technology holds great potential for discovering multigenic contributions to complex neurological disorders. These disorders are usually not inherited in a Mendelian fashion and, in many cases, may be referred to as “sporadic” cases of disease, which are ideally studied using case-control whole genome association studies of outbred populations.

The development of SNP scanning technology now allows the simultaneous testing of more than a million genetic variants, enabling initial studies into the genetics of complex disorders. Specific issues related to the technology of SNP association studies, including sample size, SNP density, and data analysis methods, are reviewed more extensively elsewhere (Craig and Stephan, 2005; Dunckley et al. 2006). Here we will discuss how the technology has been applied to neurological disorders and significant findings that have resulted.

Numerous neurological disorders have been examined using high-density, whole genome SNP association studies. These include Alzheimer’s disease (Coon et al. 2007; Reiman et al. 2007), Parkinson’s disease (Fung et al. 2006; Maraganore et al. 2005), multiple sclerosis (International Multiple Sclerosis Genetics Consortium et al. 2007), amyotophic lateral sclerosis (Dunckley et al. 2007; van Es et al. 2007; Schymick et al. 2007), bipolar disorder (Wellcome Trust Case Control Consortium, 2007; The GAIN Collaborative Research Group et al. 2007; Baum et al. 2007), and ischaemic stroke (Matarin et al. 2007).

In the majority of these diseases, it is striking that there are no single allele variants that account for the bulk of disease risk. The notable exception is that of Alzheimer’s disease, where whole genome SNP association studies confirm that the apolipoprotein E (ApoE) locus confers the largest genetic susceptibility to AD. However, when individuals are grouped and analyzed according to their ApoE genotypes, multiple SNPs within the GRB2 associated protein 2 (GAB2) gene have been reported to be significantly associated with AD risk in carriers of the ɛ4 allele of APOE (Reiman et al. 2007). These findings await further confirmation. However, this study provides a glimpse into what SNP based genotyping technology offers for the future in terms of uncovering interactions between multiple genetic loci that lead to an aggregate genetic risk for disease.

Association studies in other diseases usually identify few loci with high odds ratios (ORs) for disease. In this regard, Alzheimer’s disease is now clearly an exception among complex human diseases. The typical result appears to be identification of multiple loci with ORs less than 2. For example, a recent study in amyotrophic lateral sclerosis identified a dozen loci reproducibly associated with disease (Dunckley et al. 2007). However, no locus exceeded a 2-fold OR. Importantly, the multiple loci that were identified highlight distinct functional categories of genes. The most prominent among these groups are genes involved in neurite outgrowth. For example, a SNP within the anaplastic lymphoma kinase gene (ALK) was associated with ALS with an OR of 1.57. Independent studies showed that ALK is critical for pleiotrophin-mediated axonal regeneration in spinal motor neurons and is necessary for neuroprotection in response to glutamate excitotoxicity (Mi et al. 2007). These observations, coupled with genetic association to sporadic ALS, suggest that variations in the ALK gene could have subtle functional consequences that alter the axonal dynamics of motor neurons, ultimately leading to the development of ALS.

This study in ALS also identified candidate SNPs associated with clinical subclasses of ALS that differ in site of symptom onset, age at onset, and gender-based differences in disease onset. With further validation and confirmation, these SNPs could provide the basis for presymptomatic risk assessment to determine individuals at risk for not only developing ALS, but also for developing specific forms of ALS. This information could then be used to direct differentially treatment options. Importantly, this type of analysis highlights the direction that SNP based diagnostics and risk assessment strategies are headed to impact disease treatment approaches before initial symptoms occur. This may be a particularly important approach in neurodegenerative diseases where substantial neuronal cell loss and pathology has already occurred by the time a clinical diagnosis is made. Treatments are likely to be more effective prior to this advanced cell loss.

The use of SNP association studies for identifying multigenic contributions to complex disease risk is clearly in the early stages. The relatively low risk contributed by individual alleles will necessitate the study and analysis of significantly larger patient populations than have currently been studied. Identification of consistently reproducible genetic variants associated with complex human disease is likely to require tens of thousands of samples. Indeed, large initiatives such as the Well-come Trust Case Control Consortium and the GAIN collaborative research initiative are making the first important steps toward the study of these larger populations. However, these efforts must be expanded upon.

The utility of identifying genetic variants for complex diseases lies in their use as a predictor of disease risk, as a diagnostic for disease, as a predictor of response to therapy, or for the identification of therapeutic targets. For example, currently genetic testing is done one gene at a time using a candidate gene approach. That is, one has a family history of a particular disease for which a common genetic variant is known, such as cystic fibrosis, and can be tested for the presence of that variant within their genome. This information can then be used to guide life decisions, such as reproductive choices or exercise and eating habits in instances of other disorders. However, most human disease is sporadic and multigenic. Risk for these diseases cannot be diagnosed using traditional approaches. SNP analysis can be performed on a genome-wide scale in large case-control association studies of outbred populations to identify all of the genetic variants that contribute simultaneously to a specific disease. These variants can then be packaged into a prognostic test to predict an individual’s overall genetic risk for developing a given disease. These prognostic tests would be most effective when coupled to an effective therapeutic or prevention strategy. However, such a test to assess disease risk could have a significant impact on human health, even in the absence of a specific therapy because environmental influences, which are modifiable, also affect the development and course of disease. For example, high cholesterol diets or smoking have been shown to increase the risk of some forms of cancer and may also be an important factor in the development of Alzheimer’s disease. Knowing that you are at heightened genetic risk for these disorders could motivate significant lifestyle changes. In this way, as the analysis of complex diseases continues to evolve, the ease of use and the versatility of SNP genotyping for identifying relevant genetic variants, such as point mutations, deletions, and insertions, will lead to significant advances in human health.

### Summary

**Figure 1:**
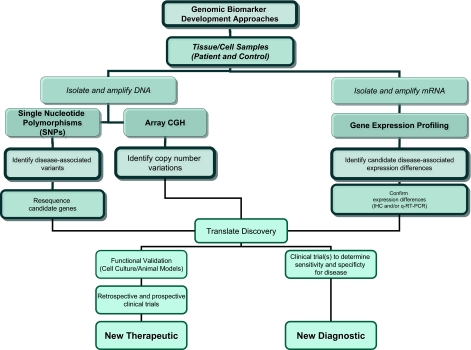
Work flow for genomic biomarker discovery in neurological diseases. Outlined are approaches using both DNA-based and RNA-based technologies. Ultimately these are discovery approaches that then must be translated into clinical application through subsequent validation efforts and confirmatory clinical trials.

Figure 1 provides a flow diagram illustrating the interconnected components of the various genomic technologies for the identification of neurological disease biomarkers. These biomarkers can be used as diagnostics or, potentially, as therapeutic targets around which treatments could be directed. Importantly, these are complementary technologies, each providing unique information about the genetic and molecular factors contributing to the disease. However, these technologies though overlapping in their utility also have unique uses. For example, SNP-based screening, and to a lesser extent aCGH, are the likely the preferred options when trying to identify biomarkers that can be used in presymptomatic risk assessment. In contrast, gene expression profiling will only show differences when some component of the disease has already manifest itself and is functionally altering the physiology of the cells or tissues. As such, gene expression profiling can provide unique diagnostic signatures or can be used to identify underlying molecular changes associated with disease. These molecular factors may serve as targets for therapeutic development. A comprehensive genomic biomarker discovery strategy will utilize each of these technologies.

## Proteomics

Proteomics refers to the systematic study of the expression, structure, and function of the proteins within cells. Ever improving technologies now permit the simultaneous detection and quantitation of several thousand proteins in normal and disease tissues and approaches for identifying post-translational variants are beginning to come to the fore. For an extensive technological review of proteomics, the reader is directed to several excellent recent review articles (Tannu and Hemby, 2006; Butterfield et al. 2006; Andrade et al. 2007). The goal of this review is to highlight important findings that demonstrate the technological potential that proteomics holds for the discovery of biomarkers and therapeutic targets in neurological disorders. Brief technological descriptions will be given where appropriate. Advances in the application of genomics to biomarker identification have led to important insights into the pathogenesis of neurological diseases, as previously described. However, genomic analysis alone will not suffice as a method to fully characterize neurological disease. Expression of messenger RNA and the encoded protein are not universally correlated. Differences in stability and turnover between mRNA and protein contribute to this weak gene-protein association. Additionally, proteins contain numerous post-translational modifications of high functional significance that cannot be inferred from mRNA expression. The proteins themselves must be analyzed directly (Rohlff, 2000). Thus, accurate monitoring of disease onset and progression requires current proteomic technologies for careful detection of protein signatures and post-translational modifications, in addition to the aforementioned genomic approaches to biomarker discovery.

Proteomics is an invaluable instrument in the search for novel disease biomarkers because current technology provides the means to describe the expression of thousands of proteins in whole cell or biofluid samples (Kinoshita et al. 2006). Proteomic analysis consists of two general steps: (1) fractionation of the complex protein mixture and (2) identification and quantitation of the separated proteins. Fractionation is usually accomplished using 2-dimensional (2-D) gel electrophoresis, liquid chromatography (LC), or both (Papasssotiropoulos et al. 2006). Proteins within the simplified mixture are typically identified using a mass spectrometry (MS) based approach, such as matrix assisted laser desorption/ionization (MALDI)-time of flight (TOF) MS or MALDI-quadrupole TOF-tandem MS (MS-MS).

Two dimensional gel electrophoresis is the most widely used separation method. The 2-D descriptor refers to the two separation steps (dimensions) involved: separation of the proteins based on isoelectric point followed by separation based on protein molecular weight. In the first dimension, the protein mixture is applied to a polyacrylamide gel with a pH-gradient and an electric potential. Proteins then accumulate at their isoelectric point. In the second dimension, the gel containing the protein spots separated by charge are denatured with sodium dodecyl sulfate (SDS) and rotated 90 degrees. An electric potential is again applied and the proteins move through the gel at a rate proportional to their sizes. Generally, 1000–3000 proteins can be visualized per gel (Banks et al. 2000). The resolved spots are then excised from the gel and proteolytically cleaved to smaller peptide fragments so that protein identification can proceed using mass spectrometry (MS) (Kinoshita et al. 2006). This approach presents several limitations. First, it is difficult to get consistent results between different polyacrylamide gels. Differential two-dimensional fluorescence gel electrophoresis (DIGE) can be used to overcome this variability. In this technique, protein samples from both a control sample and a test sample are fluorescently labeled with Cy3 and Cy5 and run simultaneously on the same gel. The fluorescence intensities can then be measured to calculate relative protein expression in control and test samples for each spot. Due to differences in dye incorporation efficiencies, both test sample and control sample must alternatively be analyzed with both Cy3 and Cy5 dyes (reciprocal labeling). Second, many proteins that are acidic or basic, hydrophobic, or of very high (>200 kD) or low (<10 kD) molecular mass, are not conducive to separation by 2-D gel electrophoresis and are more suited to separation via liquid chromatography (LC) approaches (Papasssotiropoulos et al. 2006). In LC, the protein mixture is typically first fractionated by one or a combination of binding interactions. Then the collected fractions are separated by reverse-phase high-performance LC prior to MS analysis (Papasssotiropoulos et al. 2006).

The most common method for protein identification is matrix-assisted laser desorption/ionization time-of-flight mass spectrometry (MALDI-TOF-MS). From analysis of a single tissue section, over 1000 different proteins can be simultaneously examined. A critical issue in neurological diseases is the cellular complexity of the brain. One may first reduce this complexity by isolating a more homogeneous cellular population of interest (Kinoshita et al. 2006). To this end, single cells or a small, relatively homogenous region of tissue are isolated from a heterogeneous tissue sample using laser capture microdissection (LCM) before MS analysis. However, this approach adds substantial time and effort because the detection sensitivity of current proteomic technologies requires several hundred thousand cells as starting material for protein isolations. This is a particularly relevant limitation in neurological diseases since many functionally and disease relevant processes likely occur at synapses, where proteins may be less abundant and not necessarily reflected in the cell body collected in the LCM process. It is clear that advances in sensitivity of detection thresholds and in the ability to produce homogenous sample populations are required for full utility of proteomics in understanding neurological disease.

Identification of specific proteins via MALDI-TOF-MS follows these general processes. Selected molecules are desorbed from a protein sample that has been coated with an energy-absorbing matrix whereby they become protonated and usually carry one positive charge. These desorbed ions are accelerated by a potential difference through a flight tube to strike the ion detector at the end of the tube. Due to the fact that the energy of each cation is the same, acceleration is proportional to ion mass. Data is constructed as ion density maps by plotting mass/charge signal intensities over the sample area analyzed (Johnson et al. 2006). Overall, this method allows for high-throughput quantification of global protein expression in thin, heterogeneous tissue samples and is therefore an efficient tool in the search for neurological disease biomarkers.

### Proteomic biomarker identification in neurological diseases

Several studies have utilized proteomics to discover candidate biomarkers for Alzheimer’s Disease (AD). In one study, proteins enriched in amyloid plaques in the AD brain were separated and identified using liquid chromatography and mass spectrometry, respectively. Levels of about 100 proteins were found to differ in the AD brain and Cerebral Spinal Fluid (CSF) with respect to the control brain and CSF, with the biggest changes seen in the glial fibrillary acidic protein (GFAP) (Liao et al. 2004). GFAP is an intermediate filament protein that helps to maintain the shape and mechanical strength of astrocytes. Activation of GFAP correlates well with astrocyte activation (Fuchs and Weber, 1994). Levels of this protein in AD affected individuals were observed to be 10-fold higher than in the control group, indicating significant astrocytic activation and active inflammatory processes in the AD brain. These results correlate very well with current knowledge of pathogenic mechanisms in AD and other neurodegenerative disorders, wherein inflammatory processes are thought to be an important contributor to disease progression (Liedtke et al. 1996).

Decreased levels of copper-zinc superoxide dismutase (SOD1) have also been found in AD brain samples (Gulesserian et al. 2001). SOD1 is an antioxidant that catalyzes the simultaneous oxidation and reduction of superoxide anion into hydrogen peroxide. The altered response to oxidative stress that would result from decreased SOD1 activity may contribute to neuronal cell loss in AD, a common mechanism contributing to the pathogenesis of many neurodegenerative diseases (Papasssotiropoulos et al. 2006).

The above two studies are reflective of others performed in the field in that they have not thus far yielded either significant new insights into the causes of AD or generated a reliable set of biomarkers that would be useful in the diagnosis of AD. Rather they have been useful for identifying proteins involved in pathways that have already been implicated in the disease. The overall goal is that these and future efforts at proteomics of disease-affected tissues will identify new targets for therapies that may ultimately lead to effective treatments. However, technological and technical advances will need to be made to reduce both biological and technical variability to increase the likelihood of identifying consistent changes associated with disease. Additionally, the number of proteins, as well as the associated post-translational modifications, that can be identified and quantified will need to be increased substantially. A recent study has provided an indication of the promise of proteomic technologies for biomarker identification and disease tracking and diagnosis. Lee et al. used sandwich ELISAs to identify a set of 18 proteins (from a preselected subset of 120 known signaling proteins) expressed in blood that distinguish Alzheimer’s disease patients from controls with almost 90% accuracy (Lee et al. 2007). Moreover, they were able to use this set of proteins to predict which patients with mild cognitive impairment (MCI), an early stage of cognitive impairment with high probability of progressing to AD, would advance to develop Alzheimer’s disease.

Levels of Aβ42 and total tau have shown a marked difference in AD patients (Blennow et al. 2003; Andreasen et al. 2001; Galasko et al. 1998; Hulstaert et al. 1999; Riemenschneider et al. 1996). However, total tau and Aβ42 have also been found at similar levels in the CSF of other dementias (Blennow et al. 2003; Green et al. 1999; Hampel et al. 2003; Molina et al. 1999; Kanemaru et al. 2000), but not in all (Blennow, 2003; Motter et al. 1995; Vigo-Pelfrey et al. 1995; Hulstaert et al. 1999; Sjögren et al. 2000). Additionally, increased levels of total tau have been correlated to increasing age in non-demented subjects (Hampel et al. 2003; Buerger et al. 1999; Sjögren, 2001). Thus the presence of these proteins in CSF does not appear to be specific to AD and cannot provide a reliable diagnostic biomarker.

In addition to tau protein and Aβ peptides, recent studies indicate that levels of certain phosphorylated forms of tau increase significantly in CSF of AD patients (Ewers et al. 2007; Brys et al. 2007; Kohnken et al. 2000; Arai et al. 2000; Vanmechelen et al. 2000; Hampel, 2003), but not in vascular dementia, frontotemporal dementia (Sjögren et al. 2000), Lewy body dementia, (Parnetti et al. 2001) or geriatric major depression (Buerger et al. 2003). Further, multiple studies have shown that when examining in combination the levels of phosphorylated tau, total tau and Aβ in cerebrospinal fluid (CSF), one can figure out a MCI patient’s probability for progressing to AD (Brys et al. 2007; Diniz et al. 2007; Herukka et al. 2007). These studies suggest that phosphorylated tau levels in CSF may be able to help clinically delineate AD from other forms of dementia (Blennow et al. 2003) to improve early detection and to track progression of AD.

To increase the accuracy of these proteins as a diagnostic, it would be interesting to combine this predictive set of proteins to other genetic and functional imaging biomarkers of AD to determine their combined predictive utility in a prospective longitudinal study of healthy controls and MCI patients. This may aid in the accurate early detection of AD so that therapies, which are currently in development, may be initiated prior to substantial and possibly irreparable neuronal cell loss.

It will be important to expand this analysis to a larger AD sample set, as well as to determine the specificity of these biomarkers for AD compared to other neurodegenerative diseases. Aside from this, there are at least two important implications from this finding. The first is that it is unlikely that a single gene or protein will serve as a useful diagnostic for complex disorders. More reasonably, a set of proteins or genes will be the most useful set of disease predictors. Second, this example illustrates that accurate biomarkers can be used to track disease progression from early stages to more advanced stages, in addition to being used as a molecular diagnostic for end-stage disease. This provides the hope that complex, progressive neurodegenerative disease can be presymptomatically diagnosed, thereby increasing the likelihood that therapeutic intervention will be successful.

Huntington’s Disease (HD) is a neurodegenerative disorder for which protein biomarkers have been discovered to reflect pathological status. In one notable proteomics profiling study, 18 candidate proteins were found to be differentially expressed in human plasma at different stages of HD compared to non-HD control samples. Blood samples were taken from patients in one of five disease stages: control (no disease); premanifest (positive for HD gene mutation but absent motor abnormalities); early HD; moderate HD; and advanced HD. Samples were subjected to two independent 2D Gel Electrophoresis studies and computer-assisted silver-staining imaging with digital background subtraction. Significant spots from these first studies were then identified using MS and quantified by Immunoblotting. The most promising HD biomarker finding was clusterin (apolipoprotein J), a disulfide-linked heterodimeric protein found in most mammalian tissues and bio-fluids. The presence of clusterin is associated with cytoprotection, membrane recycling, apoptosis, and response to injury (Jones and Jomary, 2002; Dalrymple et al. 2007). Notably, it is linked to immune activation, a known systemic abnormality in HD and other neurodegenerative disease. In this study, clusterin was discovered to be upregulated in the HD plasma samples, with levels following disease progression (Dalrymple et al. 2007). Overall, clusterin shows promise in acting as a biomarker of HD because it can be obtained from easily accessible biofluid, it tracks linearly with HD pathology, and it is linked mechanistically to the pathogenesis of the disease. However, because clusterin has also been reported to be upregulated at the mRNA level in AD, the specificity of this protein for HD will need to be tested further (Dunckley et al. 2006b).

**Figure 2:**
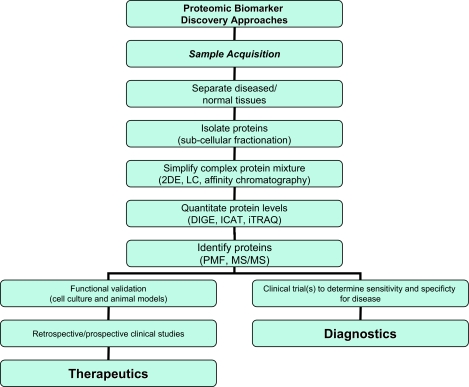
Work flow for proteomic biomarker discovery in neurological diseases. Abbreviations used are 2DE (2-dimensional gel electrophoresis), LC (liquid chromatography), DIGE (2-D fluorescence difference gel electrophoresis), ICAT (isotope-coded affinity tag), iTRAQ (isobaric tagging for relative and absolute quantitation), PMF (peptide mass fingerprinting, MS/MS (tandem mass spectrometry).

Biomarkers based on proteomic discoveries are advantageous in that peripheral tissues that are easily obtained in a clinic setting, such as saliva, serum, urine, or cerebral spinal fluid can be used for testing diagnostic signatures. This will provide a critical diagnostic adjunct to clinical disease assessment and will likely help to direct individuals to the most appropriate treatment options earlier than would otherwise happen if clinical assessments are used alone. Figure 2 summarizes the workflow for the use of proteomic technologies in biomarker discovery for neurological diseases. These technologies, though advanced substantially in the last decade, need to move further to expand the capacity to interrogate a larger number of proteins, including low abundance proteins and proteins undergoing disease relevant post-translational modifications. Ultimately, proteomic technologies hold significant promise in identification of biomarkers for disease diagnosis, tracking of disease progression, and monitoring of treatment efficacy.
